# Food consumption associated with depression, anxiety and stress in students entering a public university

**DOI:** 10.1017/jns.2024.90

**Published:** 2025-01-09

**Authors:** Maria Eduarda Ribeiro José, Ivy Evangelista Costa Ramos, Taciana Maia de Sousa, Daniela Silva Canella

**Affiliations:** 1 Programa de Pós-Graduação em Alimentação, Nutrição e Saúde – Universidade do Estado do Rio de Janeiro, Rua São Francisco Xavier, 524, 12º andar, bloco D e E, Rio de Janeiro CEP: 20550-900, RJ, Brasil; 2 Instituto de Nutrição – Universidade do Estado do Rio de Janeiro, Rua São Francisco Xavier, 524, 12º andar, bloco D e E, Rio de Janeiro, CEP: 20550-900, RJ, Brasil

**Keywords:** Depression, Food consumption, Mental disorders, NOVA classification, University, 95% CI, 95% confidence interval, COVID-19, coronavirus disease 2019, DASS-21, Depression, Anxiety, and Stress Scale-21, HUPE, Pedro Ernesto University Hospital, MW, minimum wage, OR, Odds Ratio, PR4, Pró-reitoria de Políticas e Assistência Estudantis, UERJ, Universidade do Estado do Rio de Janeiro, WHO, World Health Organization

## Abstract

Cross-sectional study investigated the association of fresh or minimally processed foods and ultra-processed food consumption with symptoms of depression, anxiety and stress in students from a Brazilian public university. Undergraduate students admitted in 2022 answered an online questionnaire during their first semester. Consumption of 12 subgroups of fresh or minimally processed foods and 13 of ultra-processed foods on the previous day were investigated (affirmative answer for ≥ 5 subgroups was classified as high consumption). Depression, anxiety and stress were investigated using the DASS-21 and mild to extremely severe symptoms were grouped to be compared with individuals without symptoms. Adjusted logistic regression models estimated the *Odds Ratio* (OR) of the association between symptoms of depression, anxiety and stress (outcomes) and food consumption (exposures), with a significance level of 5%. A total of 924 students were evaluated, of whom 57.7% presented symptoms of depression, 51.9% of anxiety and 59.4% of stress. A high consumption of fresh or minimally processed foods was observed in 80.3% of the students, with a higher frequency among those without symptoms of depression, anxiety, and stress, while 38.9% showed a high consumption of ultra-processed foods, without differences according to symptoms. High consumption of fresh or minimally processed foods was associated with a lower likelihood of symptoms of depression (OR: 0.62; p=0.011), anxiety (OR: 0.58; p=0.003) and stress (OR: 0.69; p=0.043). No association was found between ultra-processed and mental health outcomes. Actions that support and encourage the consumption of healthy food in the university environment can contribute to mental health outcomes.

## Introduction

University admission coincides with the transition period between adolescence and adulthood for most students, which can generate changes in their daily routine. The challenges of this transition range from academic demands to social, affective and psychological issues, such as leaving their parents’ home, adapting to a new social environment and having greater access to alcoholic beverages and psychoactive substances.^(1)^ These factors make undergraduate students more vulnerable to the development of mental disorders.^(2)^ In addition to this scenario, the COVID-19 pandemic (*Coronavirus disease* 2019), recognised by the World Health Organization (WHO) in March 2020, impacted the lifestyle (including food consumption, with an increase in the consumption of sugary drinks, snacks, sweets and fast food and a decrease in the consumption of fruits, vegetables and water) and the mental health of the population (students included), especially because of its health and social consequences.^(3,4)^

Regarding mental health issues, the WHO has reported a more than 25% increase in cases of anxiety and depression worldwide due to COVID-19.^(5)^ Even before the pandemic, mental disorders – defined as a change in brain function caused by biological, psychological and social factors that lead to cognitive, emotional and behavioural damage – were already among the main diseases in the world.^(6)^ In 2019, almost one billion people – including 14% of the world’s adolescents – had a mental disorder, with anxiety and depression being the most prevalent conditions. Mental disorders are the main cause of disability, and people with severe mental health conditions die 10 to 20 years earlier, on average than the general population, mainly of preventable physical diseases.^(6)^ Also according to the WHO, stress is considered ‘the 21st century health epidemic’, affecting about 90% of the world’s population and, despite not a mental disorder, it is a psychiatric symptom that can cause harm to the mental health of individuals.^(6)^ The Brazilian population presents the highest prevalence of anxiety disorders in the world and the highest prevalence of depression in Latin America.^(6)^ The National Health Survey 2019 pointed out that 10.2% of the population aged 18 years or more were diagnosed with depression, which represents 16.3 million people in the country.^(7)^ The literature indicates that this mental disorder is also important among university students, with a high prevalence of symptoms of depression (51%), anxiety (85%) and stress (65%), with more than 20% experiencing severe symptoms of these conditions.^(8,9)^

Although the aetiology of mental disorders is not entirely clear, epidemiological studies have reported a relationship between dietary patterns and mental health.^(7,10–16)^ A systematic review with meta-analysis has indicated that there are bidirectional associations between ultra-processed food consumption and adverse mental health, although it is more frequent that ultra-processed food consumption be considered as the exposure variable, with a magnitude of association in cross-sectional studies varying from OR 1.44 to 1.53.^(17)^ In a cohort study, with a sample of more than 14 thousand Spanish university graduates, individuals in the highest quartile of ultra-processed food consumption had a higher risk of developing depression (HR 1.33) than those in the lowest quartile.^(18)^ A possible explanation for this is due to characteristics of ultra-processed foods that can affect mental health, such as high energy density, which can contribute to higher adiposity, and sensorial attributes.^(12)^ Ultra-processed foods become practical and accessible options, as well as hyperpalatable, which can be stimulating or comforting for these individuals.^(13)^

Ultra-processed foods are part of the university students’ dietary habits since their food consumption is characterised by high consumption of snacks, fast food, French fries, cakes, pies, sweets and sugar-sweetened beverages, which are ultra-processed foods, and low consumption of fresh or minimally processed foods, such as fruits and vegetables. Their habits are also characterised by unhealthy practices, for example, skipping breakfast and replacing dinner with a snack.^( 14,15)^

Entry into university is a period of changes that may affect students’ mental health and some practices, which highlight the relevance of monitoring mental symptoms and disorders among students and associated factors. The intention to conduct the study in the post-pandemic period was related to the fact that on some level the COVID-19 pandemic could have affected the mental health and diet of the population, then both should be aspects of concern for universities. Therefore, this study aimed to investigate the association of the consumption of fresh or minimally processed foods and ultra-processed foods with symptoms of depression, anxiety and stress in university students.

## Methods

A cross-sectional study was conducted with a non-probabilistic sample of undergraduate students from a public university in the state of Rio de Janeiro (*Universidade do Estado do Rio de Janeiro—*UERJ), who started their undergraduate studies in 2022, namely the year of return to face-to-face activities after the COVID-19 pandemic.

Data was collected online from June to October 2022 (for first-semester students) and from October 2022 to February 2023 (for second-semester students) using a self-administered questionnaire available on the Google Forms platform. All new students were invited to participate in the study, and the first strategy to encourage their acceptance was to email them an invitation. The email addresses were provided by the Office of the Dean for Student Policies and Assistance (*Pró-reitoria de Políticas e Assistência Estudantis—*PR4) at UERJ. In addition to this strategy, the offices of the undergraduate degree programmes were also prompted to send the target students an e-mail with information about the study. A social media profile was created to disseminate information about the study. Also, on the main campus of the university (the Maracanã campus), the team of researchers personally visited classrooms, attended events, went to the university restaurant and to the student unions of the different colleges, where they distributed cards with *QR* codes that directed the respondents to the survey on the Google Forms platform, and posters with this *QR code* were put up around the campus.

The eligibility criteria included: students admitted in 2022 to all undergraduate courses from UERJ, on all 12 campuses of the university, and over 18 years of age, who accepted to participate in the study.

The data collection form was structured with open and closed questions and divided into 10 blocks: 1*—*Personal data and sociodemographic characteristics, 2*—*Nutritional status and health, 3*—*Food consumption on the previous day, 4*—*Food practices, 5*—*Food and nutrition security, 6*—*Behaviours, 7*—*Non-communicable chronic diseases, 8*—*Sleep, 9*—*Depression, Anxiety and Stress and 10*—*Household goods. The design of the questionnaire was based on questions used in national health surveys and validated scales. The variables of interest in this study are derived from blocks 3*—*Food consumption on the previous day and 9*—*Depression, Anxiety and Stress, as well as variables of characterisation of the population and non-communicable diseases, present in other blocks.

The food consumption was evaluated considering the consumption on the previous day of 12 subgroups of fresh or minimally processed foods: (a) lettuce, kale, broccoli, watercress or spinach; b) pumpkin, carrot, sweet potato or okra/caruru; c) papaya, mango, yellow melon or pequi; d) tomato, cucumber, zucchini, eggplant, chayote or beetroot; e) orange, banana, apple or pineapple; f) rice, pasta, polenta, couscous or green corn; g) beans, peas, lentils or chickpeas; h) common potato, cassava, yam or yam; i) beef, pork, chicken or fish; j) fried, boiled or scrambled egg k) milk; l) peanuts, cashew nuts or Brazil/Pará nuts); and 13 of ultra-processed foods: (a) soda; b) fruit juice in a box, box or can; c) powdered refreshment; d) chocolate drink; e) flavoured yogurt; f) packaged snacks (or chips) or salty biscuits/crackers; g) biscuit/sweet biscuit, stuffed biscuit or packaged cupcake; h) chocolate, ice cream, gelatine, flan or other industrialised dessert; i) sausage, mortadella or ham; j) sliced, hot dog or hamburger bread; k) mayonnaise, ketchup or mustard; l) margarine; m) instant noodles, packaged soup, frozen lasagna or other ready-made dish purchased frozen).

Based on these items, the following variables were constructed: score for consumption of fresh or minimally processed food and score for consumption of ultra-processed foods, which corresponds to the sum of positive responses for the consumption of at least one item in each of the subgroups (it may range from 0 to 12 and from 0 to 13, respectively); and high consumption of fresh or minimally processed items and of ultra-processed items, which was equal to or greater than 5 items. The same cutoff point is adopted by main health surveys for the adult Brazilian population (National Health Survey and Surveillance System for Risk and Protective Factors for Chronic Diseases by Telephone Survey.^(7,19)^ The questionnaire was designed considering the foods most consumed by the Brazilian population according to data from the Household Budget Survey, and it was previously validated and the cutoff was in agreement with a high energetic share of the groups in the diet.^(20,21)^ Additionally, as sensitivity analyses, different cutoffs for the dichotomous variable were tested (≥ 4 items and ≥ 3 items) and also the continuous variable.

The presence of symptoms of depression, anxiety and stress was assessed using the version of the DASS-21 scale (Depression, Anxiety, and Stress Scale-21) adapted and validated for Brazilian adolescents. This version contains 21 questions with Likert-scale answer options, ranging from 0 to 3 points (0—It didn’t happen to me this week, 1—It happened to me a few times this week, 2—It often happened to me this week, 3—It happened to me most of the time this week), according to the severity or frequency of negative emotional states experienced during the most recent week. The study variables were constructed from the sum of the answers to the items that compose each of the three subscales—depression, anxiety and stress—and each may range from 0 to 21 points. Since DASS-21 is a shorter version of the 42-item DASS, the final score of each subscale is multiplied by two and classified for symptoms into normal, mild, moderate, severe and extremely severe. For depression, the score was: 0–9 normal, 10–13 mild, 14–20 moderate, 21–27 severe and 28–42 extremely severe. For anxiety, the score was: 0–7 normal, 8–9 mild, 10–14 moderate, 15–19 severe and 20–42 extremely severe. For stress, the score was: 0–14 normal, 15–18 mild, 19–25 moderate, 26-33 severe and 34–42 extremely severe.^(22)^ Finally, mild, moderate, severe and extremely severe symptoms were grouped to be compared with individuals without symptoms, considering that any symptom, even if mild, should be looked at carefully.^(23)^ Additionally, as a sensitivity analysis, mild was grouped with individuals without symptoms.

The covariables used in the study were: age (grouped in 18–22, 23–29, 30–71 years), gender (cis woman, cis man, other), race/colour (white, mixed-race/brown, black, Asian), form of admission to university (reservation of places/quotas from the entrance exam, open competition through the entrance exam, other, later grouped into quota and non-quota students), study centre (biomedical, education and humanities, social sciences and technology and science), living arrangement (lives alone; lives with family members, not necessarily parents; lives with other people who are not family), maternal education (no education/ incomplete and complete elementary school/incomplete high school, complete high school, incomplete/complete higher education and further or did not know), family income (up to 1 minimum wage—MW, 1–2 MW, 2–5 MW, 5–10 MW, more than 10 MW; 1 MW at 2022 was equivalent to US$ 450), alcohol use (does not make use or makes use), perception of health (very good or good, regular, poor or very poor or don’t know/don’t want to answer), medical diagnosis of depression (does not received or received) and prescription of medicines for depression (does not received or received).

The quota policy is a social inclusion policy that aims to promote equity, reduce social injustices and guarantee access and retention at university.^(24)^ The quota system promotes admission via entrance exams based on economic (income), racial (black and mixed-race/brown) and educational criteria (origin from public schools), among others,^(25)^ so it can be considered a variable for social characterisation.

The answers to the questionnaire retrieved from Google Forms were exported to an electronic spreadsheet and imported into Stata/SE software version 16.0 (Stata Corp., College Station, United States) for all statistical analyses.

Descriptive analyses were made using absolute and relative frequencies for categorical variables and mean for continuous variables, with respective 95% confidence intervals (95% CI). There were significant differences based on the comparison between 95% CI values. The absence of overlap between the intervals was assumed as a significant difference, considering the significance level of 5%.

In addition, *Odds Ratio* (OR) was estimated by crude and adjusted logistic regressions (according to gender, health perception, age, family income, medical diagnosis of depression and prescription of medicines for depression) to analyse the association between the presence of symptoms of depression, anxiety, or stress (outcomes) and food consumption (exposure). The adjustment variables were selected based on the literature and their significance in the models. Significant differences were found, based on the evaluation of the p-value, which was considered significant when less than or equal to 0.05.

The study was approved by the Research Ethics Committee of the Pedro Ernesto University Hospital (HUPE/UERJ) (CAAE: 54239621.4.0000.5259). An Informed Consent Form was presented at the beginning of the online questionnaire and the participants could only continue the survey after reading and accepting it, thus being aware that all data would be used only for research purposes.

## Results

Among a total of 4,751 students beginning their undergraduate studies in 2022, 1,111 (23.4%) agreed to participate and 924 (83.2%) students were eligible. Most were aged 18 to 22 years (50.1%), cis women (62.3%), white (52.5%), non-quota students (69.9%), living with their family members (88.1%), whose maternal education was reported as incomplete/complete higher education or further (38.1%), and the family income of 31.9% ranged from 2 to 5 minimum wages. Most students reported the consumption of alcoholic drinks (61.3%) and had a perception that their health was very good or good (51.9%). Among the participants, 35.9% were from the Education and Humanities Centre which includes courses in visual arts, biological sciences, physical education, geography, history journalism, literature, pedagogy, psychology and public relations, 29.9% from the Social Sciences Centre, 29.2% from the Technology and Science Centre and 15% from the Biomedical Centre, which indicated a reasonable spread among the courses and centres. Finally, 25.4% of the students reported a previous medical diagnosis of depression and 21.7% a previous prescription of medicines for depression (Table 1).

**Table 1.** Characteristics of the sample of university students. Rio de Janeiro, 2022

Variables	N	%	95% CI
Age			
18–22 years	453	50.1	46.8–53.4
23–29 years	234	25.9	23.1–28.8
30–71 years	217	24.0	21.3–26.9
Gender			
Cis Woman	576	62.3	59.2–65.4
Cis Man	295	31.9	29.0–35.0
Other^*^	53	5.7	4.4–7.4
Race/colour			
White	485	52.5	49.3–55.7
Mixed-race/brown	235	25.4	22.7–28.3
Black	194	21.0	18.5–23.7
Asian	10	1.1	0.6–2.0
Form of admission			
Quota student	278	30.1	27.2–33.1
Non-quota student	646	69.9	66.9–72.8
Study centre			
Biomedical	138	15.0	12.8–17.4
Education and humanities	331	35.9	32.9–39.1
Social sciences	183	19.9	17.4–22.6
Technology and science	269	29.2	26.4–32.2
Living arrangement			
Lives alone	90	9.8	8.0–11.9
Lives with family members, not necessarily parents	811	88.1	85.8–90.0
Lives with other people who are not family	20	2.2	1.4–3.3
Maternal education			
No education/ incomplete and complete elementary school/incomplete high school	284	30.7	28.1–34.1
Complete high school	280	30.3	27.4–33.6
Incomplete/complete higher education and further	352	38.1	35.0–41.3
Did not know	8	0.9	0.4–1.7
Family income			
Up to 1 minimum wage (MW)	103	11.2	9.3–13.3
From 1–2 MW	181	19.6	17.2–22.3
From 2–5 MW	295	31.9	29.0–35.0
From 5–10 MW	154	16.7	14.4–19.2
More than 10 MW	191	20.7	18.2–23.4
Alcohol use			
No	358	38.7	35.6-41.9
Yes	566	61.3	58.1–64.4
Perception of health			
Very good or Good	479	51.9	48.6-55.1
Regular	356	38.5	35.4-41.7
Poor or very poor	73	7.9	6.3–9.8
Don’t know/don’t want to answer	16	1.7	1.1–2.8
Medical diagnosis of depression			
No	689	74.6	71.7–77.3
Yes	235	25.4	22.7–28.3
Prescription of medicine for depression			
No	723	78.3	75.5–80.8
Yes	201	21.7	19.2–24.5

* Other: trans man + non-binary person + prefer not to inform + other.

Regarding mental health, 57.7% presented depression symptoms, 51.9% anxiety symptoms and 59.4% stress symptoms. It is noteworthy that about 20% of the students were identified as having extremely severe depression and anxiety (Table 2). In general, the frequency of symptoms for depression, anxiety and stress was higher among women, younger and lower income students, and those with a worse perception of health. The description of the frequency of symptoms according to the characteristics of university students is presented in the Supplementary material.

**Table 2.** Frequency of depression, anxiety and stress in university students according to the severity of symptoms. Rio de Janeiro, 2022

Variables	N	%	95% CI
Depression			
Normal	390	42.3	39.1–45.4
Mild	96	10.3	8.6–12.5
Moderate	157	17.0	14.7–19.6
Severe	86	9.3	7.6–11.4
Extremely severe	195	21.1	18.6–23.9
Anxiety			
Normal	444	48.1	44.8–51.3
Mild	73	7.9	6.3–9.8
Moderate	142	15.3	13.2–17.8
Severe	80	8.7	7.0–10.7
Extremely severe	185	20.0	17.6–22.7
Stress			
Normal	375	40.6	37.5–43.8
Mild	217	23.5	20.9–26.3
Moderate	146	15.8	13.6–18.3
Severe	99	10.7	8.9–12.9
Extremely severe	87	9.4	7.7–11.5

There was high consumption of fresh or minimally processed foods (≥ 5 items) in 80.3% (95% CI 77.6–82.7) of the sample, and it was most frequently reported by students without symptoms for depression (with 76.2%; 95% CI 72.6–79.8 vs. without symptoms 85.9%; 95% CI 82.1–89.0), anxiety (with 74.8%; 95% CI 70.9–78.7 vs. without symptoms 86.3%; 95% CI 82.7–89.2) and stress (with 77.0%; 95% CI 73.5–80.6 vs. without symptoms 85.1%; 95% CI 81.1–88.3). Similarly, the mean score for fresh or minimally processed foods was higher among students without depression (with 6.4%; 95% CI 6.2–6.6 vs. without symptoms 7.2%; 95% CI 6.9–7.4), anxiety (with 6.3%; 95% CI 6.0-6.5 vs. without symptoms 7.2%; 95% CI 7.0–7.4) and stress (with 6.5; 95% CI 6.2–6.7 vs. without symptoms 7.1; 95% CI 6.9–7.4). Regarding ultra-processed foods, 38.9% (95% CI35.8–42.0) had high consumption and the mean score was 4.5 (95% CI 4.3–4.7); however, there were no differences for the presence of symptoms for high consumption or for the mean score in the study sample (Table 3).

**Table 3.** Frequency of high consumption and average score of consumption of fresh or minimally processed foods and ultra-processed foods by incoming university students according to the presence of symptoms for Depression, Anxiety and Stress. Rio de Janeiro, 2022

Variables			Depression				Anxiety				Stress			
	Total		Yes^*^		No		Yes^*^		No		Yes^*^		No	
Consumption of ≥5 items	%	95% CI	%	95% CI	%	95% CI	%	95% CI	%	95% CI	%	95% CI	%	95% CI
Fresh or minimally processed foods	80.3	77.6–82.7	76.2	72.6–79.8	85.9	82.1–89.0	74.8	70.9–78.7	86.3	82.7–89.2	77.0	73.5–80.6	85.1	81.1–88.3
Ultra-processed foods	38.9	35.8–42.0	38.8	34.6–42.9	39.0	34.2–43.9	40.4	36.0–44.8	37.2	32.8–41.8	40.4	36.3–44.6	36.5	31.8–41.6
Score of consumption	Mean	95% CI	Mean	95% CI	Mean	95% CI	Mean	95% CI	Mean	95% CI	Mean	95% CI	Mean	95% CI
Fresh or minimally processed foods	6.7	6.6–6.9	6.4	6.2–6.6	7.2	6.9–7.4	6.3	6.0–6.5	7.2	7.0–7.4	6.5	6.2–6.7	7.1	6.9–7.4
Ultra-processed foods	4.5	4.3–4.7	4.4	4.2–4.7	4.5	4.2–4.8	4.5	4.2–4.8	4.5	4.2–4.7	4.5	4.2–4.8	4.4	4.1–4.8

* Levels mild to extremely severe.

The high consumption of fresh or minimally processed foods was associated, in models adjusted by sociodemographic and perception of health, with a lower likelihood of depression (OR: 0.62; p=0.011), anxiety (OR: 0.58; p=0.003) and stress (OR: 0.69; p=0.043). The same associations were identified with the additional adjustment for medical diagnosis of depression and the use of medicines for depression, except for stress (OR: 0.72; p=0.077). For ultra-processed foods, there was no association between high consumption and the mental health outcomes analysed. However, despite of the lack of significant association, the values of OR were in the expected direction for anxiety and stress, but not for depression (Table 4). According to the sensitivity analyses, the association did not change when grouping individuals without and with mild symptoms in the category as reference. Additionally, the exposition high consumption of ultra-processed was tested considering different cutoffs, but the associations remained non-significant (data not presented).

**Table 4.** Association between high consumption of fresh or minimally processed foods and ultra-processed foods and the presence of symptoms of Depression, Anxiety and Stress in incoming university students. Rio de Janeiro, 2022

Variables	Depression			Anxiety			Stress		
	OR	95% CI	P-value	OR	95% CI	P-value	OR	95% CI	P-value
Fresh or minimally processed foods^*^									
Crude model	0.53	0.37–0.75	<0.001	0.47	0.34–0.66	<0.001	0.59	0.42–0.83	0.003
Adjusted model 1	0.62	0.43–0.89	0.011	0.58	0.40–0.83	0.003	0.69	0.48–0.99	0.043
Adjusted model 2	0.65	0.45–0.96	0.028	0.59	0.41–0.86	0.006	0.72	0.50–1.04	0.077
Ultra-processed foods^*^									
Crude model	0.99	0.76–1.30	0.948	1.15	0.88–1.50	0.311	1.18	0.90–1.55	0.232
Adjusted model 1	0.94	0.70–1.24	0.648	1.10	0.83–1.47	0.503	1.13	0.86–1.50	0.377
Adjusted model 2	0.99	0.73–1.33	0.927	1.16	0.87–1.57	0.315	1.20	0.90–1.59	0.219

OR: Odds Ratio.

Variables used in adjusted model 1: gender, perception of health, age and family income. In the case of stress, only gender, perception of health and family income were included.

Variables used in adjusted model 2: model 1 + diagnosis of depression and prescription of medicine for depression.

* Reference category: consumption of fresh or minimally processed foods and ultra-processed foods of less than 5 items.

## Discussion

Among undergraduate students from a middle-income country, more than half of the students had symptoms of depression, anxiety and/or stress. No significant difference was found between mental health outcomes and ultra-processed foods, neither considering high consumption nor the score. On the other hand, the consumption of fresh or minimally processed foods presented an inverse association with the presence of symptoms of depression, anxiety and stress, that is, for individuals with a high consumption, there was a lower likelihood of occurrence of outcomes.

We verified a high prevalence of mental health symptoms among university students, similar to other studies,^(26–30)^ despite the relatively lower frequency of medical diagnosis of depression. The sociodemographic and health characteristics distribution were also similar to other studies.^(31–35)^ Corroborating our results, other studies have shown that food consumption by young adults and university students is characterised by high consumption of ultra-processed foods and low consumption of fresh or minimally processed foods. The changes caused by university admission, for example, stress over academic demands; social, affective and psychological issues; and assuming new responsibilities such as time management, personal budget management, and food consumption, can partially account for the food consumption profile of these students.^(1,36,37)^ However, the students had entered university in the semester when they participated in the present study; for this reason, it cannot be claimed that such changes have already impacted their mental health and food consumption practices. Data from the National Health Survey 2019 showed a prevalence of high consumption (15%) of ultra-processed foods among Brazilian adults and the elderly in general, with an even higher prevalence among individuals aged 18 to 29 years (23%).^(38)^ Based on these results, it should be noted that university admission may worsen this scenario even further.

A possible obstacle to the adoption of healthy eating practices is food prices. In Brazil, prices have changed unfavourably, considering the recommendations of the Dietary Guidelines for the Brazilian Population. A study conducted by Maia and collaborators (2020) made an analysis of the average food prices in Brazil and found that the price of ultra-processed foods has been decreasing since 2000. The researchers predicted that, if this trend continues, as of 2026, healthy foods (unprocessed or minimally processed foods and processed culinary ingredients) will become more expensive than unhealthy foods (ultra-processed foods).^(39)^ Such scenario was anticipated by the pandemic,^(40)^ leading to a decrease in the quality of the population’s diet. According to a survey conducted in Latin America and the Caribbean, 1 in 2 young people faced difficulties accessing healthy food during the COVID-19 pandemic.^(41)^

In the present study, the high consumption of fresh or minimally processed foods was most often reported by students without symptoms for depression, anxiety, and stress. In addition, students with depression, anxiety and stress had a lower average of consumption than students without these problems in the score for this food group. The association between the consumption of fresh or minimally processed foods and depression, anxiety and stress remained independent of important potential confounding factors, including variables related to sociodemographic and health aspects. This shows a possible protective role of these foods for these mental health outcomes.

For ultra-processed foods, no association was found with mental health outcomes, although other evidence pointed to this conclusion.^(18,42)^ The findings of the present study may indicate, to some extent, that ultra-processed foods are widely present in the routine of university students, regardless of their mental health condition. In this study, we used a dichotomous variable, which considers a pre-defined number of ultra-processed subgroups (5 or more items), instead of their share in the diet derived from 24 hours dietary recall and food records, which were not collected. However, in the validation study of the score used, this cut-off was associated with a high share of ultra-processed foods in the diet.^(20,21)^ Alternative cutoffs were tested as also the continuous counting, but the results were similar. This result could represent a scenario of homogeneous consumption among the individuals (median=4 items; interquartile interval: 2-6), contributing to the lack of association, which differs from the literature.

The results for fresh or minimally processed foods are in line with studies with different designs that found an inverse relationship between fruit and vegetable consumption and depression.^(43–45)^ A healthy eating pattern, such as the Mediterranean, characterised by the presence of fresh or minimally processed foods such as fruits and vegetables, whole grains, lean protein sources, nuts and legumes, and low in sugars, was associated with low incidence of depression and decreased risk of anxiety.^(46,47)^ In contrast, the Western food pattern, characterised by a high consumption of ultra-processed foods, was associated with a higher risk of anxiety.^(48–50)^ The biological mechanism that explains the inverse association between fruit and vegetable consumption and mental disorders is not yet clear; however, a possible explanation would be the amount of bioactive compounds present in these foods.^(51)^

Generally, ultra-processed foods have a poor nutritional profile, with high levels of carbohydrates, saturated fats, and energy and low levels of protein and fibre.^(52)^ Such a nutritional profile has been implicated in the prevalence, incidence and severity of depression through several complex and interacting pathways, including inflammation, oxidative stress and gut microbiome. Additionally, ultra-processed foods seemingly have the potential to replace fresh or minimally processed foods, since the literature has shown systematic increases in the share of ultra-processed foods with a concomitant reduction of fresh or minimally processed foods.^(53)^ Considering that our results show an association between the consumption of fresh or minimally processed foods with a lower occurrence of adverse mental health outcomes, it is important to reinforce actions that encourage the consumption of fresh or minimally processed foods.

Although our study has tested the high consumption of fresh or minimally processed foods and ultra-processed foods such as the exposure variable and depression, anxiety, and stress as outcomes, the bidirectionality of this association should be considered. Mental well-being seems to promote a healthy lifestyle, including healthy eating and physical activity, which in turn positively reinforces the maintenance of a healthy lifestyle. On the other hand, the absence of these practices can lead to a decrease in mental well-being, which, in turn, reduces healthy lifestyle practices in a vicious cycle.^(54)^ Previous studies have reported an association between depression and unfavourable health behaviours, such as addiction to alcohol, tobacco and other substances, as well as a sedentary lifestyle and unhealthy eating habits.^(55–57)^

In view of the high consumption of ultra-processed foods instead of fresh and minimally processed foods among university students, as well as the relationship of this consumption with mental health outcomes, public health actions and policies are necessary to reverse this scenario. Importantly, the university is a favourable environment for the implementation of effective strategies of food and nutrition education; however, a continuous effort is necessary to ensure that such actions are not only informative but also able to promote healthier food choices and increase the mental health of students. In this sense, university restaurants may potentially favour the adoption of healthy eating practices. By assessing the impact of implementing UERJ’s university restaurant on students’ eating practices, researchers found that its availability on campus has helped to improve the food choices of the students that use the restaurant most frequently.^(58)^

The present study presents some limitations. The main one is the fact that the sample analysed is not representative of the participants who entered university in 2022. Although all of them had been invited to participate, approximately 20% answered the questionnaire. In general, this distribution corresponds to that observed in the university, however, the proportion of students investigated admitted at the university by the quota policy was lower (around 30% against 45%). In addition, the sample was not random; all students had been invited, only those who were willing to answer participated. Another limitation is that residual confounding cannot be discarded. In relation to food consumption, the screeners were used, which does not offer data about the total energy consumption of the university students, which could be used in the adjusted models. Additional analyses we ran including BMI and the medical diagnosis of hypertension and diabetes as adjustments, but it did not change the results. However, the analyses were adjusted for important sociodemographic information such as gender, age, family income, and also for variables related to health in general (self-perception of health) and to depression (medical diagnosis and prescription of medicine).

Despite the limitations, the study analysed a considerable amount of university students, compared to other studies that investigated the same type of population,^(59–63)^ with diverse sociodemographic and course profiles. The study was conducted shortly after the return to face-to-face classes and activities, after the period of the COVID-19 pandemic. Thus, it was an opportunity to describe phenomena that seem to have been affected by the pandemic.^(22)^ Finally, robust and validated tools were used. The first one, to assess the symptoms of depression, anxiety and stress, was adapted and validated for the population of Brazilian adolescents in order to be clearly understood by the students, and easy and quick to apply.^(22)^ The food consumption screening tools were also validated for the Brazilian population,^(21)^ are aligned with the Dietary Guidelines for the Brazilian Population^(64)^ and have been applied in national surveys.^(7,19)^ Despite the measure of the current and not the habitual consumption, the screening tools used can be considered an innovative aspect of the study since in 2024 a guideline proposed by the World Health Organization suggested their usage to monitor diets globally.^(65)^

The results of this study indicate a significant portion of undergraduate students experience mental health problems, with relevant frequencies of depression, anxiety and stress symptoms. The food consumption was characterised by high consumption of fresh or minimally processed foods and ultra-processed foods, but the consumption of this last group tended to be homogenous in the sample. High consumption of fresh or minimally processed foods was associated with a lower likelihood of depression, anxiety and stress. However, in our study, there was no significant association between the consumption of ultra-processed foods and mental health outcomes. In this context, universities become important spaces for the development of strategies to encourage health promotion and adequate and healthy eating, in addition to strengthening policies for student support and permanence. Therefore, the present study reinforces the relevance of carrying out actions to encourage the consumption of fresh or minimally processed foods and to discourage the consumption of ultra-processed foods. In this context, the promotion of a healthy university food environment can also contribute to good mental health.

## Supporting information

José et al. supplementary material José et al. supplementary material

## Supplementary material

The supplementary material for this article can be found at https://doi.org/10.1017/jns.2024.90
