# Supplementary material for: Food consumption associated with depression, anxiety and stress in students entering a public university
Source: J Nutr Sci. 2025 Jan 9;14:e3. doi: 10.1017/jns.2024.90 (PMC11811848; doi:10.1017/jns.2024.90)
Supplement: José et al. supplementary material [file S2048679024000909sup001.docx]

| SUPPLEMENTARY MATERIAL – Frequency of symptoms for depression, anxiety, and stress according to characteristics of university students. Rio de Janeiro, 2022. | | | | | | | | | |
| --- | --- | --- | --- | --- | --- | --- | --- | --- | --- |
| **Variables** | **Depression** | | | **Anxiety** | | | **Stress** | | |
|  | **N** | **%** | **95% CI** | **N** | **%** | **95% CI** | **N** | **%** | **95% CI** |
| ***Age*** |  |  |  |  |  |  |  |  |  |
| 18-22 years | 278 | 61.4 | 56.8-65.8 | 252 | 55.6 | 51.0-60.2 | 279 | 61.6 | 57.0-66.0 |
| 23-29 years | 143 | 61.1 | 54.7-67.2 | 133 | 56.8 | 50.4-63.1 | 153 | 65.4 | 59.0-71.2 |
| 30-71 years | 102 | 47.0 | 40.4-53.7 | 86 | 39.6 | 33.3-46.3 | 105 | 48.4 | 41.8-55.0 |
| ***Gender*** |  |  |  |  |  |  |  |  |  |
| Cis Man | 159 | 53.9 | 48.2-59.5 | 113 | 38.3 | 32.9-44.0 | 148 | 50.2 | 44.5-55.9 |
| Cis Woman | 339 | 58.9 | 54.8-62.8 | 330 | 57.3 | 53.2-61.3 | 362 | 62.8 | 58.8-66.7 |
| Other or did not answer | 36 | 67.9 | 54.2-79.1 | 37 | 69.8 | 56.1-80.7 | 39 | 73.6 | 60.0-83.8 |
| ***Quota student*** |  |  |  |  |  |  |  |  |  |
| Yes | 177 | 63.7 | 57.8-69.1 | 159 | 57.2 | 51.3-62.9 | 175 | 62.9 | 57.1-68.4 |
| No | 357 | 55.3 | 51.4-59.1 | 321 | 49.7 | 45.8-53.5 | 374 | 57.9 | 54.0-61.7 |
| ***Living arrangement*** |  |  |  |  |  |  |  |  |  |
| Lives alone | 52 | 57.8 | 47.3-67.6 | 52 | 57.8 | 47.3-67.6 | 57 | 63.3 | 52.9-72.7 |
| Lives with family members | 470 | 58.0 | 54.5-61.3 | 419 | 51.7 | 48.2-55.1 | 477 | 58.8 | 55.4-62.2 |
| Lives with other people who are not family | 11 | 55.0 | 33.1-75.1 | 8 | 40.0 | 21.0-62.6 | 14 | 70.0 | 46.6-86.2 |
| ***Family income*** |  |  |  |  |  |  |  |  |  |
| Up to 1 minimum wage (MW) | 11 | 68.8 | 42.4-86.8 | 12 | 75.0 | 48.2-90.6 | 12 | 75.0 | 48.2-90.6 |
| From 1-2 MW | 56 | 64.4 | 53.7-73.8 | 55 | 63.2 | 52.6-72.7 | 59 | 67.8 | 57.3-76.8 |
| From 2-5 MW | 118 | 65.2 | 57.9-71.8 | 101 | 55.8 | 48.5-62.9 | 115 | 63.5 | 56.3-70.2 |
| From 5-10 MW | 268 | 59.7 | 55.1-64.1 | 234 | 52.1 | 47.5-56.7 | 269 | 59.9 | 55.3-64.4 |
| More than 10 MW | 81 | 42.2 | 35.6-49.6 | 78 | 40.8 | 34.1-48.0 | 94 | 49.2 | 42.2-56.3 |
| ***Alcohol use*** |  |  |  |  |  |  |  |  |  |
| No | 206 | 57.5 | 52.3-62.6 | 179 | 50.0 | 44.8-55.2 | 207 | 57.8 | 52.6-62.8 |
| Yes | 328 | 58.0 | 53.8-62.0 | 301 | 53.2 | 49.0-57.3 | 342 | 60.4 | 56.3-64.4 |
| ***Perception of health*** |  |  |  |  |  |  |  |  |  |
| Very good or good | 224 | 46.8 | 42.3-51.3 | 195 | 40.7 | 36.4-45.2 | 248 | 51.8 | 47.3-56.2 |
| Regular | 243 | 68.3 | 63.2-72.9 | 216 | 60.7 | 55.5-65.6 | 234 | 65.7 | 60.6-70.5 |
| Poor or very poor | 56 | 76.7 | 65.6-85.1 | 58 | 79.5 | 68.6-87.3 | 55 | 75.3 | 64.1-83.9 |
| Don’t know/don’t want to answer | 11 | 68.8 | 42.4-86.8 | 11 | 68.8 | 42.4-86.8 | 12 | 75.0 | 48.2-90.6 |
| ***Medical diagnosis of depression*** |  |  |  |  |  |  |  |  |  |
| No | 342 | 64.0 | 59.9-68.0 | 305 | 63.5 | 59.1-67.7 | 364 | 66.3 | 62.2-70.1 |
| Yes | 192 | 36.0 | 32.0-40.1 | 175 | 36.5 | 32.3-40.9 | 185 | 33.7 | 29.9-37.8 |
| ***Prescription of medicine for depression*** |  |  |  |  |  |  |  |  |  |
| No | 376 | 70.4 | 66.4-74.1 | 328 | 68.3 | 64.0-72.3 | 390 | 71.0 | 67.1-74.7 |
| Yes | 158 | 29.6 | 25.9-33.6 | 152 | 31.7 | 27.7-36.0 | 159 | 29.0 | 25.3-32.9 |
| *Levels mild to extremely severe. | | | |  |  |  |  |  |  |
